# Dietary sodium restriction reduces blood pressure in patients with treatment resistant hypertension

**DOI:** 10.1186/s12882-023-03333-9

**Published:** 2023-09-19

**Authors:** Bodil G. Hornstrup, Nikolai Hoffmann-Petersen, Thomas Guldager Lauridsen, Jesper N. Bech

**Affiliations:** 1 University Clinic in Nephrology and Hypertension, Gødstrup Hospital and Aarhus University, Hospitalsparken 15, Herning, DK-7400 Denmark; 2 Department of Medicine, Gødstrup Hospital, Herning, Denmark

**Keywords:** Ambulatory blood pressure monitoring, Dietary sodium restriction, Sodium sensitivity, Endothelial function

## Abstract

**Purpose:**

Patients with treatment resistant hypertension (TRH) are at particular risk of cardiovascular disease. Life style modification, including sodium restriction, is an important part of the treatment of these patients. We aimed to analyse if self-performed dietary sodium restriction could be implemented in patients with TRH and to evaluate the effect of this intervention on blood pressure (BP). Moreover, we aimed to examine if mechanisms involving nitric oxide, body water content and BNP, renal function and handling of sodium were involved in the effect on nocturnal and 24-h BP. Also, measurement of erythrocyte sodium sensitivity was included as a possible predictor for the effect of sodium restriction on BP levels.

**Patients and methods:**

TRH patients were included for this interventional four week study: two weeks on usual diet and two weeks on self-performed sodium restricted diet with supplementary handed out sodium-free bread. At the end of each period, 24-h BP and 24-h urine collections (sodium, potassium, ENaC) were performed, blood samples (BNP, NOx, salt blood test) were drawn, and bio impedance measurements were made.

**Results:**

Fifteen patients, 11 males, with a mean age of 59 years were included. After sodium restriction, urinary sodium excretion decreased from 186 (70) to 91 [51] mmol/24-h, and all but one reduced sodium excretion. Nocturnal and 24-h systolic BP were significantly reduced (− 8 and − 10 mmHg, respectively, *p* < 0.05). NOx increased, BNP and extracellular water content decreased, all significantly. Change in NOx correlated to the change in 24-h systolic BP. BP response after sodium restriction was not related to sodium sensitivity examined by salt blood test.

**Conclusion:**

Self-performed dietary sodium restriction was feasible in a population of patients with TRH, and BP was significantly reduced. Increased NOx synthesis may be involved in the BP lowering effect of sodium restriction.

**Trial registration:**

The study was registered in Clinical trials with ID: NCT06022133.

## Introduction

Elevated blood pressure is the main contributor to the risk of developing cardiovascular disease (CVD) and is yearly accountable for more than 10 million premature deaths worldwide [1, 2]. One subgroup of patients with hypertension is defined by lack of blood pressure (BP) control despite three antihypertensive medications, one of them being diuretic, all in optimal dosing [3]. The condition is referred to as treatment resistant hypertension (TRH). Patients with TRH are at particular risk of CVD and target organ damage [4, 5]. In later years of research, it has become evident, that high nocturnal BP and blunting of normal nocturnal BP decrease, defined non-dipping, are stronger correlated to the risk of CVD than high day time BP [6, 7]. There is incipient evidence of a protective effect of lowering nocturnal BP on the risk of CVD [8]. Hence, it is of interest to investigate the effect of BP lowering actions on nocturnal BP as well as 24-h BP.

Non-adherence to medication is a general problem. It has been shown in several studies that non-adherence is commonly seen in patients diagnosed with TRH [9–11]. In one clinical study, it was revealed that witnessed intake of antihypertensive medication before initiation of 24-h BP monitoring reduced the number of hypertensive patients in a population of patient suspected of TRH [12]. Hence, this method may be one way to avoid, at least to some extent, the challenge of non-adherence.

A cornerstone in treatment of patients with hypertension is lifestyle modification [13]. High sodium intake increases the risk of treatment resistant hypertension [14]. Therefore, it is important to reduce sodium intake in these patients. General population in western societies ingests an average of 10 g pr. day [15, 16]. This is despite recommendations to lower sodium intake to 6 gram/ day [17]. Evidence from clinical studies has shown decreased BP levels following sodium restriction [18, 19]. Many of the studies conducted to analyse effect of sodium restricted diet on BP, including the DASH-study, have used fully prepared and handed-out diets [19–21]. This is not easily implemented in the general population.

Dietary sodium restriction does not result in equal BP response in all individuals. Sodium sensitivity may be one of the explainable mechanisms involved in the diverse response to sodium restriction. Sodium sensitivity is a result of many different individual features, i.e. renal function, age, and sodium buffering capacity in skin, bone and endothelia [22]. Sodium has been shown to influence the endothelial function, and in-vitro studies have demonstrated, that high concentrations of sodium reduce the amount of the protective glycocalyx layer on vascular endothelial cells [23, 24]. The endothelial sodium buffering capacity has been examined in a set-up, where the erythrocyte sedimentation rate in a sodium buffer has been evaluated [25]. The erythrocytes capacity to buffer sodium was defined as erythrocyte sodium sensitivity (ESS). ESS was improved, when the erythrocytes were pretreated with a glycocalyx protector. Hence, measurement of ESS may be one way of evaluating sodium sensitivity and foresee which patients could benefit from sodium restricted diet.

In this study, we aimed to analyse if self-performed sodium restricted diet could be implemented in a group of patients with TRH. The diet was self-made, except for handed out sodium-free bread. The diet is therefore potentially implementable to general population. Moreover, we aimed to explore if the changes in nocturnal and 24-h BP in response to sodium restriction could be explained by mechanisms involving nitric oxide synthesis, water retention (extracellular water and BNP), renal function, or renal handling of sodium (ENaCɣ). Finally, we intended to analyse whether BP response to sodium restriction could be predicted by measurement of erythrocyte sodium sensitivity.

## Materials and methods

### Design

This study was designed as an interventional study. The effect of the intervention was compared to a preceding non-interventional period (control period), and the patients therefore served as their own controls. The order of non-intervention and intervention period was not randomised.

### Study setting

The study was carried out in the University Clinic in Nephrology and Hypertension and the Renal Outpatient Clinic, Gødstrup Hospital, Herning, Denmark. Patients were included from January 2018 until March 2021.

### Study population

The study included patients with treatment resistant hypertension according to the following inclusion criteria: men and women 20–70 years old, eGFR > 45 ml/min, unchanged prescriptions of antihypertensives for ≥ 3 months, and 24 h BP ≥ 130/80 mmHg performed after handed out, supervised and witnessed intake of usual antihypertensive medicine by study manager.

Exclusion criteria: Symptoms of heart failure (NYHA class 3–4), clinically relevant peripheral oedema, treatment of obstructive sleep apnoea, forced expiratory volume of 1 s < 50% of expected, antihypertensive medication that cannot be dosed once daily, pregnancy or lactation, p-albumin < 34 g/l, urine albumin/creatinine > 1000 g/mg, INR > 1.2, and coelic disease.

Withdrawal criteria: development of exclusion criteria, withdrawal of consent, or lack of compliance for attendance.

### Number of patients

14 patients had to be included, if the minimal relevant change in night-time SBP from baseline to end of intervention is 6 mmHg, SD is 8 mmHg, and a significance level of 5% and a power of 80% are used.

### Effect variables

Primary effect variable was change in nocturnal systolic BP (SBP).

Secondary effect variables were changes in 24-h and day time BP, in relative nocturnal SBP decrease, in total urinary sodium excretion, in the ratio of day/night urinary sodium excretion, in total body water and extracellular water, in urinary excretion of u-AQP2 and u-ENaCɣ, in plasma NOx (nitrite + nitrate), BNP, and sodium sensitivity (SS) (from salt blood test, SaBT).

### Procedure

Patients in the Outpatient Clinic were reviewed regarding age, renal function, and medicine prescriptions. If these were as listed in the inclusion criteria, recent home or 24-h BP measurements were evaluated. If these were above limits for normal BP and no antihypertensives had been added since, patients were regarded eligible. These patients were asked about the possibility of participating in the study at a planned control at the Renal Outpatient Clinic. If the patient was interested, an information meeting was planned, and after informed written consent was provided, screening examinations were performed (baseline). The screening consisted of health information obtained from a questionnaire, review of prescribed medications, blood tests (creatinine, sodium, potassium, total cholesterol, HDL, LDL, triglycerides, HbA1c, INR, ALT, albumin), and urine examination (u-albumin-creatinine ratio). After the above mentioned, 24-h ABPM was performed after intake of usual antihypertensive medication witnessed by the study manager, and 24-h urine collection was carried out (creatinine, sodium, potassium, urea, and albumin).

If patients fulfilled inclusion criteria after the above completed examinations, they were included for participation and started 14-day standard period with usual diet. None of the patients had received any dietary counselling regarding sodium intake prior to study participation, and they were informed not to change anything in their usual diet.

At day 11 to 14, examinations were performed. This is referred to as examination 1 (E1). At day 11, patients were weighted and lower leg circumference was measured 25 cm proximal to the medial malleolus. Patients answered questionnaire about medicine adherence, any symptoms of high or low blood pressure (dizziness, headache), bio impedance measurements were performed, and blood samples were drawn (Salt blood test, BNP, NOx, creatinine, sodium, potassium, and albumin). Also 24-h ABPM was performed. Patients collected urine for 24 h in two cans¸ the first from morning until final voiding at bedtime, the second can for eventual voiding during night-time until final voiding when getting up in the morning.

When examinations at E1 were completed, patients continued into the intervention period as described below. At day 11–14 in the intervention period, identical examinations as at E1 were performed. These examinations are referred to as E2. Measurements were performed on the same side as at E1.

After completion of these examinations, patients had completed project participation.

Planning of the examination days could diverge within a few days in order to optimal planning for the patients.

### Intervention

In the two weeks intervention period, patients were instructed in self-performed sodium restriction in the diet. They received oral and written instruction on how to perform the sodium restriction, and two kinds of sodium-free bread were handed out for the two weeks period. The written instruction included general advice on how to reduce sodium intake: avoid / limit prefabricated bread, avoid take-away and industrially processed food, avoid industrially marinated food and industrially processed cold cuts, avoid cheese, limit the use of butter, avoid biscuits and chips, and avoid having a sodium container at the dinner table. Also, the written instruction included main meal suggestions and food items to avoid. For breakfast, patients were advised to eat oatmeal, dairy products, fruits, and avoid cereals and cheese. For lunch, patients were advised to eat the handed-out bread, leftovers from dinner, fruits, vegetables, eggs (without salt), salads, and cold cuts with sodium content less than 0.6 g/ 100 g and only one slice. They were advised to avoid cold cuts with higher sodium content than 0.6 g/ 100 g, sausages, tuna, smoked salmon, and marinated herring.

For dinner, patients were advised to avoid fast food and prefabricated food, to prepare food themselves without addition of salt (also broth cubes) but to use other spices instead. If they were planning to eat pies or pizza, they were advised to prepare the dough themselves, and they were advised to fry in cooking oil instead of butter.

If patients had any questions to intervention, they were informed to contact the study manager for guidance.

### Antihypertensive medication

All medications were taken in the morning. The antihypertensive medication was dosed by the study manager into date- and weekday-marked boxes, and identical generic drugs were used for all the patients’ usual prescriptions, and all were given in the usual dosage. Medications for the whole study period were handed out in the beginning of the standard period. Any possible change in medication was registered in the patient’s study file, and patients were asked to bring their medication boxed at examination day as a control for adherence. They were also asked, how many dosings they had missed out in the preceding period.

Patients were informed to contact the study manager, if any side effects occurred.

### Oscillometric blood pressure

Twenty-four hour BP measurement and office BP were measured with an oscillometric device, A&D TM-2430 (A&D Company Ldt., Tokyo, Japan). Circumference of the upper left arm was measured and an appropriate size cuff was chosen. Office BP was measured after a minimum of 5 min rest in sitting posture. Throughout the 24-h, BP was measured every 20 min. Day- and nocturnal blood pressure was defined from the patients’ information about bedtime. The same device was used for the patient for all three measurements (baseline, E1, and E2).

### Definition of hypertension and non-dipping

Hypertension and non-dipping were defined according to the 2018 guidelines from the European Society of Cardiology (ESC) and the European Society of Hypertension (ESH) [3]. Hypertension was defined as 24 h SBP/DBP ≥ 130/80 mmHg. Non-dipping was defined as relative nocturnal decrease in SBP ≤ 10%. Defined daily doses of antihypertensive medication were classified using the ATC/DDD kit from WHO.

### Sodium sensitivity

ESS was measured using Salt Blood Test Mini from K-EDTA blood. Blood samples were drawn and within 15 min. 50 μL blood was mixed with 50 μL Na + cocktail (CARE Diagnostica Laborreagenzien, Voerde, Germany) and shaken by hand. Within 15 min. the mixture was reshaken thoroughly and filled into hematocrite tubes using gravity forces. The tubes were placed vertically in tube holders. The length of the supernatant was measured and read after exactly 60 min. ESS was then calculated based on standards according to the manufacturer.

Erythrocyte sodium sensitivity (ESS) was measured from SaBT using the following two equations: (value from SaBT, mm/21.4)*100 for men, and (value from SaBT, mm/26.1)*100 for women. From ESS, patients are categorized as low, average, or high sodium sensitive based on ESS < 80, 80–120, or > 120, respectively [26].

### Biochemical analyses

The Department of Clinical Biochemistry, Gødstrup Hospital, Denmark analysed plasma (creatinine, sodium, potassium, total cholesterol, HDL, LDL, triglycerides, HbA1c, INR, ALAT, albumin) and urine (creatinine, sodium, potassium, urea, and albumin) samples using routine methods. eGFR was calculated using the CKD-EPI equation.

Blood samples were centrifuged and plasma separated immediately after being drawn from the patients. Samples were kept frozen at -20 º C or -80 º C.

BNP were analysed at the Department of Clinical Biochemistry, Gødstrup Hospital, Denmark by chemiluminescent microparticle immunoassay. Minimal detection level was 2.89 pmol/l.

Urine samples were kept frozen at -20 º C until assayed. U-ENaCɣ was analysed using radioimmunoassay as previously described [27]. Antibodies against synthetic peptides for ENaCɣ were raised in rabbits.

NOx were determined using chemoluminiscence technique provided by Zysense Nitric Oxide Analyzer (NOA 280i) (Zysense, Frederick, Colorado, USA). This method has previously been described [28].

Osmolality (urine and plasma) was determined by freezing point depression (A_2_O Advanced Automated Osmometer, Advanced Instruments, Norwood, Massachusetts, USA).

### Body water content

Body Composition Monitor, Fresenius Medical Care was used for non-invasive measurement of body water content and extracellular water as previously described [29]. Measurements were made with the patients in prone position after 2 min rest. Only measurements with data quality above 90% were accepted, and measurements were repeated until three consecutive measurements met this quality standard. The mean value of the three measurements was used for data analyses.

### Statistical methods

All statistical analyses were made by the authors using IBM SPSS statistics version 22.0 (IBM Corp.; Armonk, NY, United States). All data were tested for normality and variance equality. In all of the analyses, statistical significance level was set to *p* < 0.05. Normally distributed continuous data were presented as means with standard deviation (SD) in parenthesis.

Sodium-to-potassium-ratio was calculated by dividing 24-h excretion of sodium with 24-h excretion of potassium, both expressed as mmol/24-h.

Unpaired data were tested for difference using t-test, and Mann-Whitney’s test was used if data were not normally distributed. These data are presented as median values with minimum and maximum value in brackets [min;max]. Continuous paired variables were tested for difference using the paired t-test, and Wilcoxon’s test was used if paired continuous data were not normally distributed. Categorical variables were listed as percentages with total number in parenthesis. To test for differences on a binary paired variable, McNemar’s test was used.

Correlation analyses were performed to test the association between changes in BP and changes in sodium excretion and for the significance of sodium sensitivity (from salt blood test). Associations between selected variables and change in blood pressure were investigated using correlation analyses following the “intention to treat” principle. Correlation analyses were performed using Pearson’s test, if data were normally distributed; otherwise Spearman’s test was used.

Multivariate regression analyses were performed using a linear regression model to investigate the relationship between variables. The dependent variable was absolute change in SBP (24-h SBP or nocturnal SBP). The first independent variable was change in 24-h sodium excretion (absolute or relative change as nominal variable or relative change as categorical > or < 50% reduction). The second variable was either ESS1 or absolute change in NOx. In all, five analyses were performed, and the exact model is described in the results section.

## Results

### Demographics

**Fig. 1:**
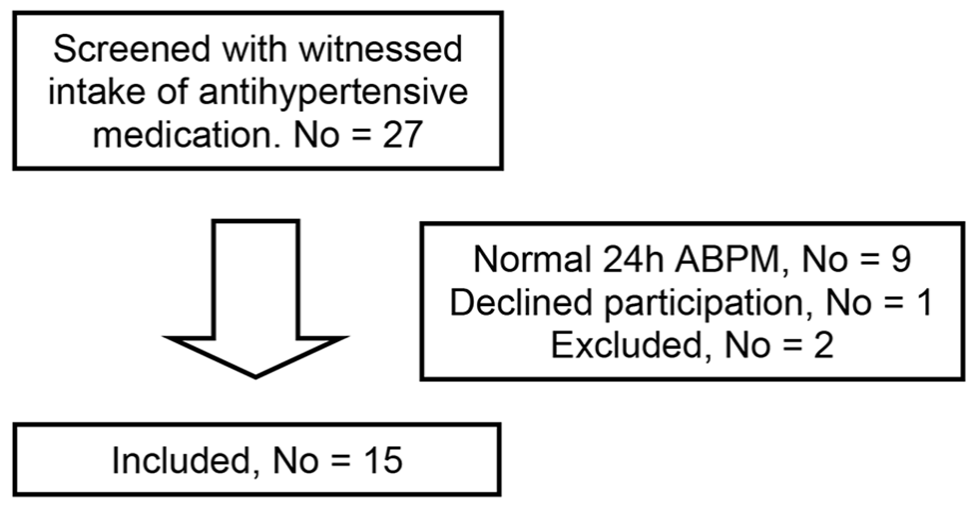
Flow chart of screening and inclusion of patients **Abbreviation**: No, number

A total of 27 patients were screened for participation. Flow chart is seen in Fig. 1. Nine of the patients had normal 24-h BP, one declined participation. Two were excluded; one of them because of acute illness within the first week of participation, the other due to lack of time to participate. There was no loss to follow-up, and 15 patients were included for intervention and for data analyses.

These 15 patients, 11 (75%) of them men, had a mean age of 59 [7] years and mean BMI 30 [4]. They had been treated for hypertension for a median of 12 years [3;30] and received a median of 5.5 [3;10] defined daily doses of antihypertensive medication. Eight patients (53%) received potassium-sparing diuretic.

Baseline 24-h systolic/ diastolic BP was 144 [12]/82 [9] mmHg. Baseline 24-h sodium excretion was 192 (86) mmol, which corresponds to 11.2 gram of salt. Baseline 24-h potassium excretion was 68 [20] mmol, which equals 2.7 gram. Baseline sodium-to-potassium ratio was 2.9 (1.3).

Information on smoking history, diabetes, and medication is shown in Table 1.

**Table 1:** Baseline characteristics of the patient population

	N = 15
Age, years, mean (SD)	59 (7)
Gender, men, No. (percent)	11 (73)
24-h urinary sodium excretion, mmol (SD)	192 (86)
eGFR, mL/min/1,73m2, mean (SD)	74 (20)
Body mass index, kg/m2, mean (SD)	30 (3)
HbA1c, mean (SD)	45 (15)
Diabtes, no. (percent)	5 (33)
Statin treatment, No. (percent)	6 (40)
Platelet inhibitor treatment, No. (percent)	1 (7)
Active smokers, no. (percent)	2 (13)
Former smokers, no. (percent)	8 (53)
Package years, median (min;max)^a^	28 (3;59)
Office systolic BP, mmHg, mean (SD)	157 (36)
Office diastolic BP, mmHg, mean (SD)	89 (16)

^a^ For active and former smokers

**Abbreviations** :BP, blood pressure; h, hour; HbA1c, haemoglobin A1c; No, number; SD, standard deviation.

The first period, from inclusion to end of examinations at E1, lasted a median of 13 days [8;17], while the second period from finishing examinations at E1 to finishing examinations at E2, lasted a median of 14 days [13;16].

Medicine prescriptions regarding antihypertensive medication were identical at E1 and E2.

### Blood pressure

As seen in Table 2, 24-h BP was unchanged from baseline to E1. From E1 to E2, all BP variables decreased (24-h, day, and nocturnal) significantly, both systolic and diastolic. There was no change in relative nocturnal BP decrease, neither in absolute values (14 vs. 13 mmHg, *p* = 0.78). This is depicted in Table 2.

**Table 2:** Data from 24-h blood pressure measurements at Baseline, Examination 1 and 2

	Baseline^a^	Examination 1	Examination 2	∆ (E1-baseline)	∆ (E2-E1)
24-h systolic BP, mmHg, mean (SD)	144 (12)	141 (13)	131 (12)	-3 (8) ^ns^	-10 (8)**
24-h diastolic BP, mmHg, mean (SD)	81 (9)	81 (10)	76 (6)	-1 (4) ^ns^	-5 (5)*
Day time systolic BP, mmHg, mean (SD)	149 (12)	145 (15)	136 (13)	-4 (10) ^ns^	-9 (9)**
Day time diastolic BP, mmHg, mean (SD)	86 (10)	85 (11)	80 (7)	-1 (1) ^ns^	-5 (6)*
Night time systolic BP, mmHg, mean (SD)	133 (16)	131 (18)	123 (14)	-2 (13) ^ns^	-8 (13)*
Night time diastolic BP, mmHg, mean (SD)	74 (11)	74 (11)	69 (8)	0 (6) ^ns^	-5 (7)*
Relative nocturnal BP decrease, percentage, mean (SD)	11 (9)	9 (12)	9 (9)	-2 (8) ^ns^	0 (8) ^ns^

^a^From 24-h blood pressure monitoring, measured after observed intake of antihypertensive medication

**Abbreviations**: BP, blood pressure; E1, examination 1; E2, examination 2; h, hour; SD, standard deviation.

Explanation of *P-*values: ^ns^= non-significant, *=<0.05, **<0.001

At E1, three (20%) had controlled hypertension, and at E2, this number was 6 (40%). This change was non-significant (*p* = 0.25). At E1 and E2, 8 and 9 patients were non-dippers, respectively.

Results were similar after exclusion of one patient, who did not reduce sodium intake.

### Analyses of 24-h urine

From baseline to E1, 24-h urinary sodium excretion remained unchanged (192 (86) mmol vs. 186 (69) mmol, *p* = 0.97). From E1 to E2, 24-h urinary sodium excretion decreased significantly by 96 mmol, as seen in Table 3. This change corresponds to approx. 5.5 g of salt. All patients but one, showed a decrease in urinary sodium excretion, and the median relative decrease for the whole population was 48% (total range − 25 to 80%).

**Table 3:** Data from 24-h urine collection at Examination 1 and examination 2

	Examination 1	Examination 2	∆ (E2-E1)	*p*-value
24-h sodium excretion, mmol (SD)	186 (70)	91 (51)	-96 (61)	0.00003
24-h potassium excretion, mmol (SD)	71 (23)	72 (12)	1 (20)	0.90
24-h sodium-to-potassium excretion (SD)	2.7 (1.0)	1.3 (0.6)	-1.4 (0.8)	< 0.0001
24-h urea excretion, mmol (SD)	387 (103)	360 (70)	-38 (95)	0.30
24-h albumin excretion, median (min;max)	14 (4;37)	9 (1;47)		0.33
Nocturnal / 24-h sodium excretion rate, median (min;max)	0.56 (0.22;1,92)	0.70 (0.26;2.37)		0.031
Nocturnal / 24-h creatinine excretion rate, mean (SD)	1.10 (0.20)	1.22 (0.23)	0.12 (0.24)	0.73

**Abbreviations**: E1, examination 1; E, examination 2, h, hour; SD, standard deviation

Also seen in Table 3, nocturnal sodium excretion rate in relation to 24-h excretion rate was significantly increased at E2 compared to E1, whereas the same analysis of creatinine was unchanged. Absolute sodium excretion decreased significantly during daytime (80 mmol, [-5; 118], *p* = 0.034), whereas the nocturnal change in excretion rate was unchanged (8 mmol [-51;79], *p* = 0.2) Sodium-to-potassium excretion more than halved from E1 to E2 (Table 3).

The absolute 24-h excretion of potassium, creatinine, urea, and albumin were all unchanged.

### Erythrocyte sodium sensitivity

At E1, mean erythrocyte sodium sensitivity (ESS) was 88 [37]. The patients were characterised as follows according to sodium sensitivity: six as low, six as average, and three as high. At E2, mean ESS was 78 (73) with 8 as low, five as average, and two as high. ESS at E1 and E2 were correlated (Pearson’s correlation coefficient 0.896, *p* < 0.0001), and the difference between them was non-significant (*p* = 0.247).

### Body water content

As seen in Table 4, patients weight and leg circumference were significantly reduced. No significant changes in fat or lean tissue mass (absolute or relative) were seen.

**Table 4:** Body weight, body water content, and lower leg circumference at Examination 1 and Examination 2

	Examination 1	Examination 2	∆ (E2-E1)	*p*-value
Body weight, kg, mean (SD)	91.9 (14.8)	90.7 (14.7)	-1.2 (1.09)	0.0005
Total body water, L, mean (SD)^a^	49.0 (5.9)	46.5 (5.3)	-2.5 (3.1)	0.007
Extracellular water, L, mean (SD)^a^	21.5 (3.0)	20.2 (2.8)	-1.3 (1.4)	0.003
Intracellular water, L, mean (SD)^a^	27.5 (5.7)	26.4 (3.0)	-1.1 (2.0)	0.04
Circumference, lower leg, cm, mean (SD)	39.5 (2.3)	38.6 (2.2)	-0.8 (1.4)	0.041

^a^Data from body composition monitoring

**Abbreviations**: E1, examination 1; E, examination 2; kg, kilo gram; L, liters; cm, centimetres; SD, standard deviation

### Blood and urinary biomarkers

eGFR did not change from E1 to E2 (74 [20] vs. 69 [22] mL/min/1,73 m^2^, *p* = 0.089).

Median value of BNP decreased significantly from E1 to E2 (6.1 [2.9;26.3] pmol/l vs. 3.9 [2.9;19.3] pmol/l, *p* = 0.008).

Mean plasma NOx level was significantly higher at E2 than at E1 (30.5 (15.4) μmol/l vs. 40.4 (19.1) μmol/l (*p* = 0.019)).

Urinary excretion rate of ENaCɣ did not change from E1 to E2 (1.02 (0.40) ng/min vs. 1.04 (0.46) ng/min (*p* = 0.85)).

### Univariate and multivariate correlation analyses

**Fig. 2:**
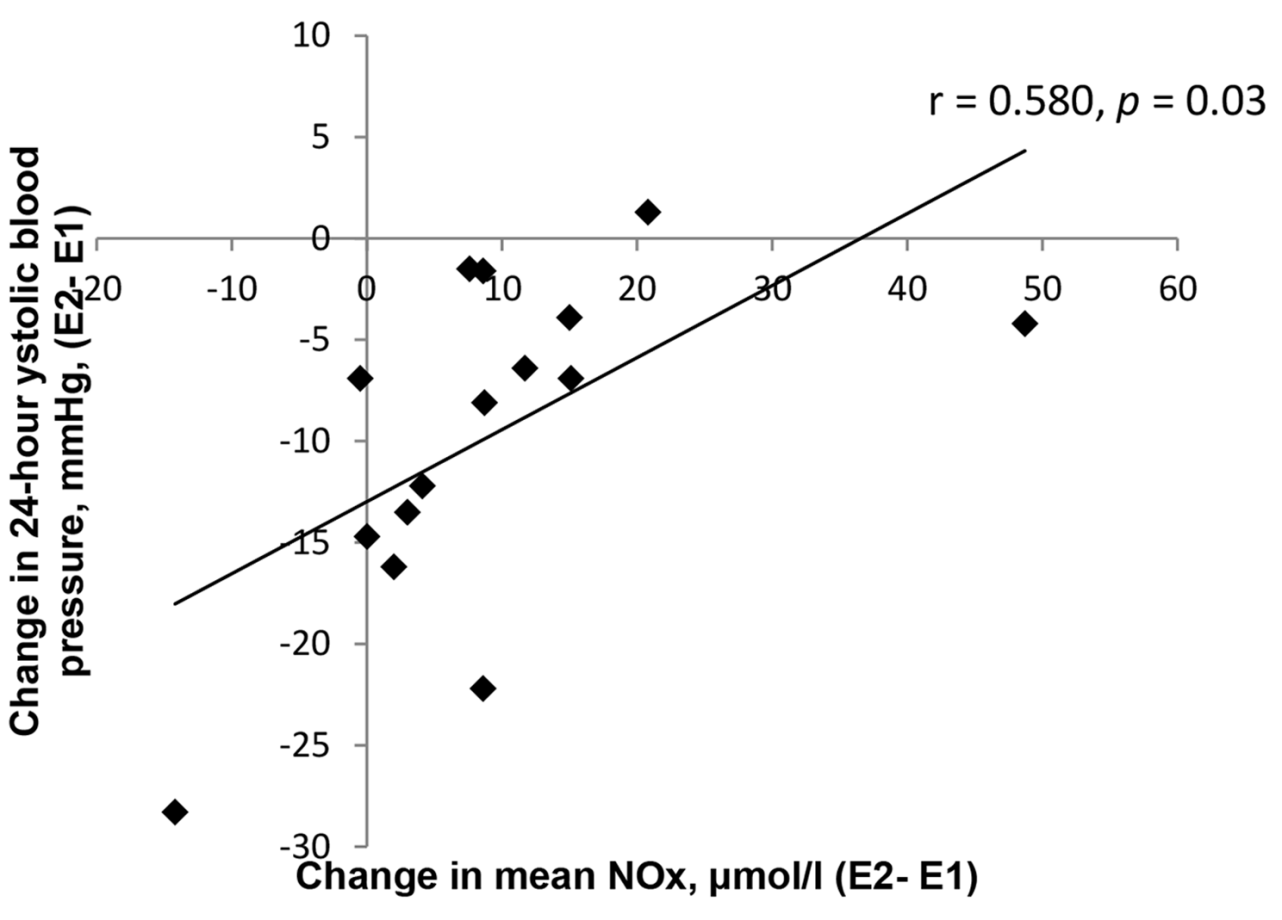
Correlation between change in 24-hour systolic blood pressure and change in NOx **Abbreviations**: E1, examination 1; E, examination 2; No, nitric oxide

Changes in BP parameters were not correlated to relative or absolute change in sodium excretion or to change in sodium-to-potassium excretion. The absolute change in 24-h SBP correlated significantly to the absolute change in NOx (r = 0.58, *p =* 0.03). This correlation is illustrated in Fig. 2. Changes in the other BP parameters were not correlated to NOx.

**Fig. 3:**
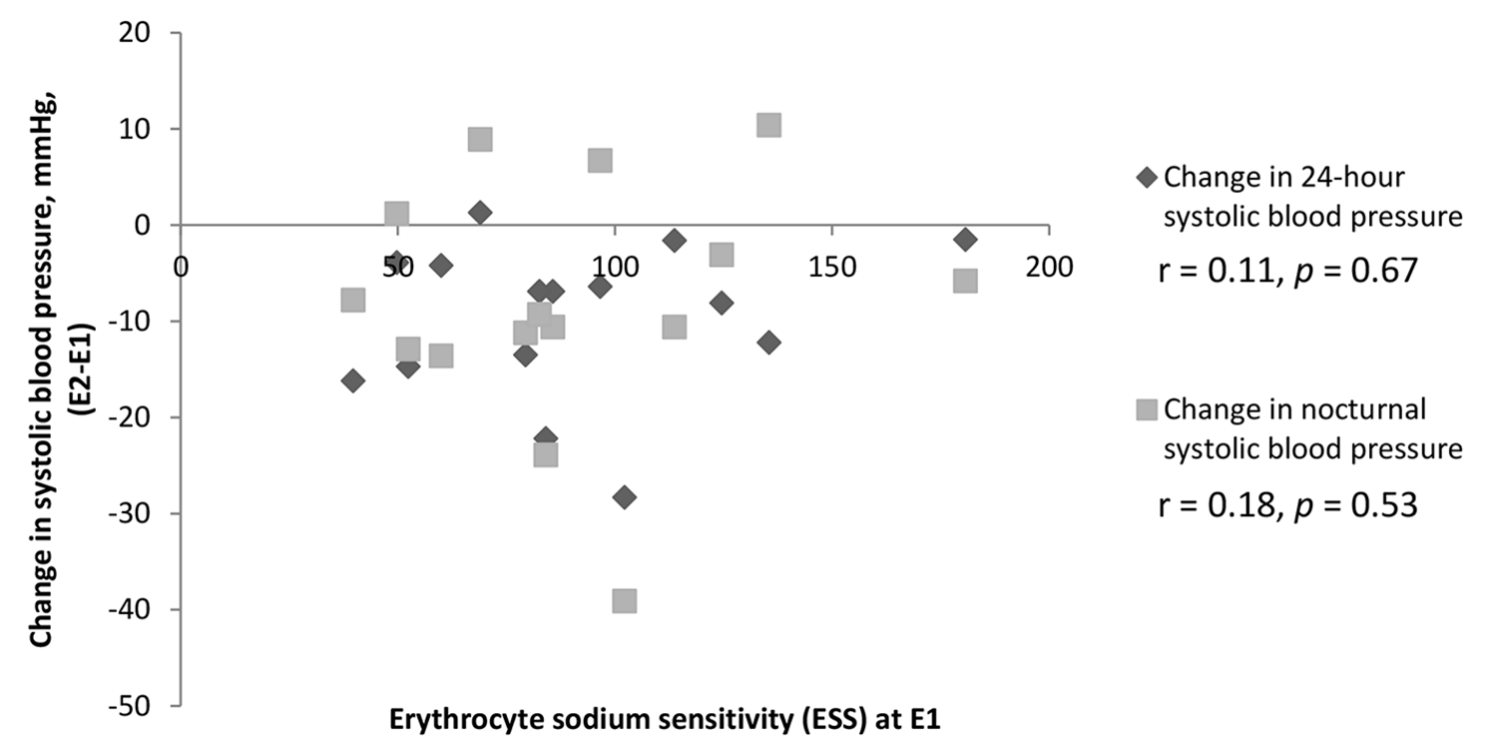
Correlation between ESS (erythrocyte sodium sensitivity) at E1 and changes in 24-hour and nocturnal systolic blood pressure **Abbreviations**: E1, examination 1; E, examination 2

There was no correlation between nocturnal BP levels and nocturnal sodium excretion (absolute, relative to day, or relative to 24-h excretion) at neither E1 nor E2. Changes in BP parameters were not correlated to ESS at E1 or to changes in ESS. The correlations between ESS at E1 and change in 24-h and nocturnal SBP are depicted in Fig. 3.

BNP was not correlated to the change in TBL, change in BP, or sodium excretion.

Multivariate regression analyses with change in BP (24-h or nocturnal SBP) as dependent variable, change in 24-h sodium excretion (absolute change as nominal variable or relative as categorical >/< 50% reduction) and ESS1 as independent variables, did not show any correlation.

Multivariate regression analysis with absolute change in 24-h SBP as dependent variable, relative change in 24-h sodium excretion and absolute change NOx as independent variables, did show an association (For the whole model: R^2^ = 0.432, *p* = 0.044 with absolute change in plasma NOx as the only significant independent variable (*p* = 0.025)).

### Side effects

Side effects were recorded in four patients: three reported new-onset dizziness at follow-up; two of them had 24-h BP reduction > 10 mmHg systolic, the third had only minor reduction in BP (1 mmHg). Mean BP change for these three patients was not different than in the population without dizziness. One patient had increased creatinine and potassium at follow-up.

## Discussion

In this population of patients with TRH, we found a significant reduction in BP parameters following dietary sodium restriction for two weeks. The change in BP was not correlated to the reduction in urinary sodium excretion or to the reduction in sodium-to-potassium excretion. Patients in this study completed dietary sodium restriction based on dietary instructions and handed-out sodium-free bread. Baseline sodium excretion for this population equalled 11 g of salt and was reduced to around 5.2 g. In this study, we used a new biochemical marker of sodium sensitivity; salt blood test. This marker could, however, not predict the effect of dietary sodium restriction on BP. Following the sodium restriction, plasma NOx levels increased, and this change correlated to 24-h SBP reduction. Patients had reduced body water content and BNP levels after sodium restriction as well.

It is well-known, that dietary sodium restriction, even modest, can lower BP [18, 19, 30]. However, in this study we included a population of resistant hypertensive patients. This population has not previously been very well-examined in this context. In 2009, Pimenta et al. presented data from 12 subjects with treatment resistant hypertension [20]. They experienced a 20 mmHg difference in 24-h SBP when comparing fully prepared handed out meal with low or high sodium intake (50 mmol vs. 250 mmol sodium intake). The BP response to sodium restriction in that study is greater than in our study, which may be related to difference in sodium load. In our study, the intervention was based on the patients’ usual diet. Therefore, the 10 mmHg reduction in 24-h SBP from our data is probably more applicable to the general population.

Sodium restriction based on fully prepared sodium-restricted or sodium-free meals is not easy to adapt in the general population. A recent study by Riis et al. demonstrated that introduction of sodium-free bread to the diet of healthy families reduced sodium excretion by 0.6 g (1.5 g of salt) in adults [31]. In our study, sodium restriction was based on oral and written information and sodium-free bread. Compared to the effect of sodium-free bread in families from the previous study, patients in the present study managed to cohere to other changes in diet in addition to the handed-out sodium-free bread. The latter is not possible for the patients to achieve unless patients bake their own bread. Hence, the daily challenge in sodium restricted diet is still obvious in western countries, where about 70% of sodium is added outside home (i.e. industrially prepared food, restaurants etc.) [32].

The response to sodium loading in humans is highly variable [22]. Sodium sensitivity is defined as BP increase as a response to sodium load [33]. There is, however, many different definitions of sodium sensitivity, and there is no simple way to allocate individuals as sodium sensitive or not. Oberleithner et al. described a method of measuring erythrocyte sodium sensitivity (ESS) [26]. They demonstrated that ESS was improved, when pre-treating the erythrocytes with a glycocalyx protector [25]. In this study, we did not succeed in demonstrating, that ESS was related to changes in BP as a response to sodium restriction. Therefore, we could not use this marker to predict the effect of sodium restriction. There is a biologically variation in ESS. Hence, a larger population may be needed in order to use ESS as a predictor of the response to sodium restriction.

Only 20% of patients in this study was characterised as *sodium sensitive* according to this test. This is a low number if we compare to findings from previous studies of hypertensive individuals using other criteria for diagnosing sodium sensitivity [25, 34, 35]. There was a non-significant tendency towards decreased ESS at follow-up. This may be a sign of improved sodium buffer capacity. We only included patients for two weeks of sodium restrictive intervention. ESS is a marker of sodium buffering capacity. Thus, a longer intervention period may result in changes in ESS. Moreover, the intervention was self-performed and therefore only semi-controlled. A more controlled intervention for may result in ESS changes for the whole population.

The study from Pimenta et al. presented evidence of reduced volume load (lower BNP and higher renin activity) during sodium restriction [20]. In our study, we, as Pimenta et al., also found evidence of reduced body water content by bio impendance measurements and analyses of BNP. In our study, the change in body water content did not correlate to the change in sodium excretion. In the body, sodium is stored in water containing compartments but also in skin and bones without supplementary water [36]. This storage may serve as a buffer when sodium intake is high, and excretion of sodium from this storage would not lead to decrease in water storage.

In our study, we did, however, demonstrate significantly lower BNP levels at follow-up. In a community study of a population without known heart failure, it was demonstrated that levels of BNP could predict the risk of developing cardiac disease, even at low levels of BNP [37]. Consequently, reduction of BNP in our population may implicate reduced risk of developing cardiovascular disease.

Previous studies have related high nocturnal sodium excretion to high nocturnal BP and lack of nocturnal BP decrease [38]. As a proof of this idea, non-dipping pattern have been restored when reducing sodium load [39].

In our study, the relative nocturnal excretion rate of sodium of 24-h excretion rate increased after sodium restriction, whereas the relative nocturnal creatinine excretion of 24-h excretion remained unchanged. The decrease in 24-h sodium excretion was mainly attained though reduced daytime excretion, as nocturnal excretion was unchanged. As a consequence, the excretion rate of sodium was not correlated to nocturnal BP levels. Therefore, we could not demonstrate evidence of Guytons pressure natriuresis theory directly in our patients [40]. The preceding studies have been conducted on essential hypertensive patients, not on patients with TRH. Hence, the regulatory mechanisms may be altered in our population.

From experimental animal models, it is proposed that the capability to store sodium non-osmotically actively is one of the traits that defines sodium-resistant individuals [41]. Also, the glycocalyx in skin and arteries have been shown to be involved in the buffering capacity of sodium [42]. Oberleitner et al. found that sodium overload damaged the protective function of the endothelial glycocalyx, thereby allowing sodium to enter and alter the endothelial cells [24, 43].

The endothelial cells elicits local vasodilation by producing NO [44]. This ability is reduced in patients with hypertension. This has further negative consequences on its function caused by exposure to high sodium intake. The effects of sodium are seen directly on NO synthesis, but also on the ability of the endothelial cells to generate a vasodilating response to NO [43, 45].

The analyses of NOx in this study measure primarily nitrate. The effector molecule of the NO-system is NO. This molecule is generated by serial degradations of nitrate to nitrite and, finally, to NO [46]. We found higher NOx levels after sodium restriction in our patients. The change in NOx was correlated to the reduced 24-h SBP level even when adjusting for change in sodium excretion. Hence, in this population of treatment resistant hypertensive patients the effect on BP may in part be explained by NO dependent mechanisms. This may be a combination of both enhanced endothelial NO production and improved ability of the endothelial cells to respond to NO when sodium load is diminished.

In our study, we found no change in urinary excretion of potassium or urea. Urinary potassium excretion has been shown to correlate to dietary intake of potassium-containing food [47]. Hence, the finding in this study may be evidence of unchanged dietary intake of potassium containing foods (e.g. vegetables), and changes in potassium intake therefore cannot explain the BP changes as a response to dietary sodium restriction. The aforementioned study by Pimenta et al. found an increase in urinary potassium excretion despite fixed potassium in the diet [20]. The increased potassium excretion can be related to the increased aldosterone levels and renin activity, which was demonstrated in that study. All patients in that study received thiazide-diuretics and ACE-inhibitors / AII antagonists. In our study, we did not investigate elements of the RAA system. More than half of our patients received potassium sparing diuretics, which may blunt any effect of the increasing activity in RAAS after introduction of sodium restriction.

Our patients reduced sodium-to-potassium excretion significantly. This was based on reduced sodium excretion, since potassium, as mentioned above, was unchanged. The change was, however, not correlated to changes in blood pressure. Previous population studies do not agree upon, whether blood pressure levels and sodium-to-potassium-ratio are correlated [48, 49]. Hypertensive patients have other risk factors for development and maintenance of high blood pressure. Consequently, the effect of sodium-to-potassium excretion may not stand independently as a risk marker.

We analysed urinary excretion of ENaCγ as a marker of renal handling of sodium. Excretion rate of ENaCγ remained unchanged. Hence, transport of sodium from the tubular lumen across the epithelial sodium channel in the principal cell to the intracellular space in the distal part of the nephron did not seem to be affected by changes in sodium intake.

Aldosterone induces increased surface expression of ENaC in the endothelial cells, and it induces increased activity of ENaC in the principal cell leading to retention of sodium and loss of potassium [50]. Reduced sodium intake increases aldosterone levels, thereby increasing ENaC activity followed by increased urinary excretion of the γ-fraction. In animal studies, blocking of the mineralocorticoid receptor antagonist have been shown to influence this compensatory response to sodium depletion and reducing the expected increase in excretion rate of ENaCγ [51]. More than half of the patients in the present study received potassium-sparing diuretics, which may explain the lack of changes in the ENaCγ excretion despite reduced sodium loading.

All patients had hypertension at baseline. However, BP decreased non-significantly from baseline examination to E1, therefore three of the patients had controlled hypertension at E1. We screened patients after witnessed intake of antihypertensive medication to ensure that we included true resistant hypertensive patients. Moreover, we handed out the medication for the study period to heighten the compliance. Hence, we did some effort to accommodate non-adherence to medication, but we could not fully exclude the existence of it. Previous studies have shown, that despite efforts to increase hypertensive patients’ adherence to medication, many patients still remain non-adherent [10].

Patients had, despite long term diagnosis of hypertension, a high baseline sodium excretion. Hence, either lifestyle modification talks had not been performed and/ or patients had been resistant to this. Neither way should health care professionals be very aware of this challenge.

It is interesting that we observed decreased urinary sodium excretion, decreased body water content, and a decrease in BP parameters, and that these observations do not seem to be correlated. However, power calculation was based on the change in nocturnal BP, and lack of correlation between the latter mentioned effect variables may be related to statistical power.

### Strengths and limitations

We used 24-h BP measurement, both at inclusion and at both examinations. We used the same equipment for all examinations on the same patient.

Patients completed 24-h urine collection at baseline in order to control for the Hawthorne effect. Sodium excretion at baseline and E1 did not differ significantly; hence, patients did not implement sodium restriction before entering the intervention period.

Patients completed sodium restriction for only two weeks in this study. This is a short period, and the magnitude of the sodium reduction may be influenced by high motivation because of the short period of time. Studies with longer follow-up are warranted to explore the possibilities of implementing longer term sodium restriction.

The study was conducted as an interventional study. We did not include a control group. Therefore, we are not able to control for change in diets or behaviour in general (adherence to medication, exercise e.g.) for patients included in a study analysing blood pressure.

Moreover, we did not randomise the intervention as we expected that patients would be challenged in changing back to normal diet after a period of dietary sodium restriction. Patients received usual antihypertensive medication throughout the study period. Therefore, we avoided analyses on elements of the RAA system. It did, however, give us a view of the add-on effect of sodium restriction in addition to usual antihypertensive treatment.

We used 24-h urine collection for assessment of sodium intake. This is a robust method, but it is, however, affected by other circumstances. Among them weekly and/or monthly rhythmically changes in endogenous regulation of sodium homeostasis. However, Walsers analyses showed that T_½_ for sodium balance, when sodium intake is changed, is 21 h [52]. Hence, if a new steady intake is applied for 4 days, total body sodium content and sodium excretion has reached a new steady state. Our patients were included for intervention for 14 days.

## Conclusion

In a population of 15 treatment resistant hypertensive patients, we demonstrated that self-performed dietary sodium restriction could be implemented safely. After two weeks of sodium restriction, BP and urinary sodium excretion were reduced significantly. Increased nitric oxide synthesis may be involved in the BP lowering effect of sodium restriction alongside with reduced body water content.

## Data Availability

Data are available upon request to corresponding author.

## Abbreviations

ABPM: Ambulatory blood pressure monitoring
BMI: Body mass index
BNP: Brain natriuretic peptide
BP: Blood pressure
CV: Cardiovascular
CVD: Cardiovascular disease
DBP: Diastolic blood pressure
eGFR: Estimated glomerular filtration rate
ENaCɣ: Endothelial Sodium Channel ɣ
ESS: Erythrocyte sodium sensitivity
h: Hour
HbA1c: Hemoglobin A1c
NO: Nitric oxide
RAAS: Renin angiotensin aldosterone system
SaBT: Salt Blood test
SBP: Systolic blood pressure
TRH: Treatment resistant hypertension
